# *Ascaris* and *Escherichia coli* Inactivation in an Ecological Sanitation System in Port-au-Prince, Haiti

**DOI:** 10.1371/journal.pone.0125336

**Published:** 2015-05-01

**Authors:** David Berendes, Karen Levy, Jackie Knee, Thomas Handzel, Vincent R. Hill

**Affiliations:** 1 Department of Environmental Health, Rollins School of Public Health, Emory University, Atlanta, Georgia, United States of America; 2 Emergency Response and Recovery Branch, Division of Global Health Protection, Centers for Disease Control and Prevention, Atlanta, Georgia, United States of America; 3 Environmental Microbiology Laboratory, Waterborne Disease Prevention Branch, Division of Food, Waterborne, and Environmental Diseases, Centers for Disease Control and Prevention, Atlanta, Georgia, United States of America; ContraFect Corporation, UNITED STATES

## Abstract

The goal of this study was to evaluate the microbial die-off in a latrine waste composting system in Port-au-Prince, Haiti. Temperature data and samples were collected from compost aged 0 – 12+ months. Samples collected from compost bin centers and corners at two depths were assessed for moisture content, *E*. *coli* concentration, and *Ascaris* spp. viability. Center temperatures in compost bins were all above 58 °C, while corner temperatures were 10 – 20 °C lower. Moisture content was 67 ± 10% in all except the oldest compost. A 4-log reduction in *E*. *coli* was observed over the first sixteen weeks of composting at both locations and depths, after which *E*. *coli* was undetectable (LOD: 142 MPN g^-1^ dry weight). In new compost, 10.4% and 8.3% of *Ascaris* eggs were viable and fully embryonated, respectively. Percent viability dropped to zero in samples older than six weeks. These findings indicate that the Haitian EcoSan composting process was effective in inactivating *E*. *coli *and *Ascaris* spp. in latrine waste within sixteen weeks. This study is one of the first to document efficacy of an ecological sanitation system under field conditions and provides insight into composting methods and monitoring for other international settings.

## Introduction

Worldwide, almost 1.8 billion people have gained access to improved sanitation over the past two decades. However, 2.5 billion people, spread disproportionately across the developing world, still lack this basic human right [1].

Sanitation systems in the developed world typically utilize water-based flush toilets that require substantial investment in pipes, sewers, and wastewater treatment plants. However, in less developed areas, decentralized sanitation systems provide a cost-effective, feasible alternative and may have advantages that reduce environmental impact and increase sustainability. Certain types of decentralized sanitation technologies, such as ecological sanitation (EcoSan) systems, are designed to recycle waste material [2]. EcoSan, developed in the late 1990s, was designed to “close the loop” between sanitation and agriculture by recycling nutrients from composted human waste into agricultural fertilizer. EcoSan systems reduce nutrient and water waste, as well as water consumption, through various methods. EcoSan projects have been implemented by governmental and non-governmental organizations (NGOs) throughout the world in the last decade, with successful case studies in Uganda, Denmark, Finland, and Germany [2, 3].

One additional benefit of EcoSan systems is the fertilizer generated from composted human waste, which must be treated to ensure safety in handling and use on food crops [4, 5]. EcoSan toilets, including urine-diverting composting toilets (UDTs) and other small-scale sanitation facilities, utilize methods such as aerobic or anaerobic digestion, vermicomposting (use of worms to process compost), filtration, and desiccation (natural or with addition of ash or lime for pH adjustment) to inactivate pathogens in waste [3, 6]. In composting latrines, organic material is added to the solid waste and the compost process inactivates pathogens and digests the material into fertilizer. Heat inactivation at temperatures of 55–65°C provides the simplest and often most practical method for inactivating pathogens in compost [4, 7].

To date, the few evaluations of composting latrine projects in field settings have focused on inactivation of fecal indicator organisms in EcoSan compost [4, 8–14]. Fecal indicator bacteria are used as a proxy for pathogens, and are also used to standardize the monitoring of microbial inactivation processes [7, 15]. However, these bacterial indicator organisms may not be good surrogates for parasitic or viral pathogens, which are more persistent in treatment systems and in the environment. Instead, helminthes, such as *Ascaris* spp., which are more resistant to desiccation and heat, provide a more conservative measure of composting treatment efficacy [15]. *Ascaris* spp. are commonly studied in soil and compost as indicators of the maximum potential survival of pathogenic microorganisms in these matrices [13, 16–18].

In this study, we measured both *E*. *coli* and *Ascaris* spp. to evaluate microorganism survival in latrine waste and compost from EcoSan toilets in Haiti constructed and maintained by Sustainable Organic Integrated Livelihoods (SOIL), an NGO based in Port-au-Prince and Cap-Haitien. The goal of this study was to evaluate microbial reductions in a centralized EcoSan composting system.

## Materials and Methods

### Study Site Description

Haiti was one of a few countries to experience a decrease in access to improved sanitation between 1990 and 2010, from 26% to 17% [1], and the January 12^th^, 2010 earthquake considerably worsened the sanitation situation in the capital of Port-au-Prince. At the time of this study, SOIL had installed over 200 EcoSan latrines in several sites in Port-au-Prince, designed as UDTs with urine diverted to a plastic collection container and feces caught in barrels beneath the toilet. During typical operation, users add a handful of sugarcane husk on top of their feces after each use to limit odor and facilitate aeration. The sugarcane husk also acts as an organic material to aid in the composting process and is widely available in Haiti.

**Fig 1:**
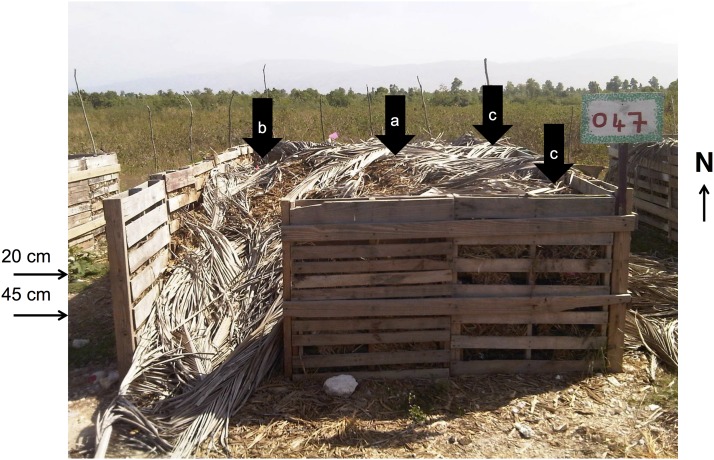
Compost bin sampling locations. Example of a SOIL compost bin, showing sampling locations and depths. Samples were collected in all bins from the a) center and b) northwest corner. Samples were also collected from the c) northeast and southeast corners in the bin with newly added latrine waste (Bin 1). The southwest corner was not sampled due to a lack of compost at the opening.

Once full, barrels are replaced and consolidated 2–3 times per week at a central composting site near the Cité Soleil region of Port-au-Prince, generating fertilizer to be sold after a period of 9–12 months. This centralized or ‘off-site’ composting site (in contrast to in-latrine models) contains 20–25, 3- x 6-m compost bins (example shown in Fig 1) spaced approximately 2 m apart. The bin walls are made from wooden forklift pallets stuffed with sugarcane husk, and one corner of each bin is left open to allow access. The floors of the bin are earthen, with no material lining the bottom. Each new bin is filled with untreated latrine waste/husk mixture for a period of two weeks, to a height of approximately 1–1.5 m. After the filling period, an additional 5–10 cm of sugarcane husk (usually with palm fronds) is placed atop of the compost pile to maintain heat and protect from wind, thus no fecal material is present at the surface of the compost pile. The composting process is static (i.e. not mixed) for 6 months, although over the first 2–3 months of composting, approximately 190 L of urine collected from the toilets is added to the bin over multiple watering sessions to increase moisture [19]. Compost is generally well-distributed over the bin space, with a slight mound forming in the center and less compost in the corner open to access.

### Sample Collection

Permission was obtained from SOIL prior to collecting samples at the composting site. A total of 37 compost samples were collected, representing waste material and compost of different ages:

- *Untreated latrine waste samples* were new waste/husk mixtures, collected to characterize baseline compost prior to entering the bin;
- *Repeat samples* were compost material over its first 14 days in the bin, collected to characterize early stage organism die-off (Bin 1); and
- *Cross-sectional samples* were compost material from bins ranging in age from 2 to 96 weeks. Two types of cross-sectional samples were collected: ‘intermediate’ (2–44 week old compost, Bins 2–10) and ‘final’ (48–96 week old compost, ready to be sold as fertilizer and no longer in bins).

Sampling occurred between June 20 and July 6, 2012 in Haiti. Table 1 presents details of numbers of each type of sample collected.

**Table 1:** Sampling Frequencies and Locations.

Bin	Age of compost	Bin Condition	# of sampling events	Bin Sampling Location
N/A	0 days	Untreated, actively being filled	6	N/A*
1	1, 3, 8, 10, 14 days	‘Intermediate’, most recently filled	5	5 center, 6 corner
2–10	2, 4, 6, 8, 12, 16, 20, 24, 44 weeks	‘Intermediate’	9	1 center, 1 corner
N/A	48–96 weeks	‘Final’, ready for sale	2	N/A**
TOTAL			22	

*Untreated samples were taken directly from latrine waste collection containers prior to filling compost bin

**Final compost samples were collected directly from bags prior to sale

The untreated latrine waste samples were collected from multiple barrels being added to the bin on a single day to generate a composite sample; six composite samples were collected over the two week period and results of these samples were averaged to generate a baseline value. Untreated latrine waste samples were collected in single 500-mL sterile Nalgene bottles using a sterile, large stainless steel spoon that had been rinsed with a 10% bleach solution, dried, rinsed with 70% ethanol, and dried again.

To capture potential spatial heterogeneity within bins, samples from Bin 1 (repeat samples) and Bins 2–10 (intermediate-aged cross-sectional samples) were collected at two depths (20 cm and 45 cm) from both the center and corner of the bin. These depths were chosen to represent approximately one-third and one-half of the total depth of the compost itself (excluding the 5–10 cm of husk added to the top after filling). Center and corner designations were made by dividing the bins into nine equal sections and selecting from the center section and a corner section. Corners where the bin entrance was located were not selected for sampling because they contained insufficient compost. The northwest corner was selected in each bin sampled, with the exception of Bin 1, where the northwest, northeast, and southeast corners were each sampled twice, approximately one week apart, on a rotating basis over the two-week period (Fig 1).

For sample collection in both the center and corner of the bin, a ~50-cm deep hole was dug by hand at each site—in the center portion of the bin for the center sample and ~25 cm from each wall of the bin for the corner sample. Hands were double-gloved and covered with a plastic sheet up to the elbow for digging. After digging the hole, the plastic sheet was removed and a new one was used for collection at 45 cm to avoid cross-contamination. After the 45-cm sample was collected, the plastic sheet was removed, as well as one layer of gloves, and a new plastic sheet was used for the 20-cm sample collection. Samples were deposited into sterile polypropylene Nalgene bottles. Two sterile 125-mL bottles—one at each depth—were filled with sampled compost material for *E*. *coli* testing. A sterile 500-mL bottle was filled with a pooled sample comprised of equal proportions of compost collected from the two depths for *Ascaris* spp. analysis, with the goal of collecting 300 g of compost per bin location. Bottles were filled to maximum capacity to minimize head space and sealed tightly.

‘Final’ samples were collected from storage sacks at SOIL’s office in Port-au-Prince. A sterile 500-mL Nalgene bottle was used to scoop and collect these samples directly.

All samples were brought to the Laboratoire National de Santé Publique (LNSP; Haitian National Public Health Laboratory) within 4 hours of collection and immediately placed at 4°C until shipping or processing. *E*. *coli* analysis was carried out on the day of collection at LNSP. *Ascaris* spp. viability testing and moisture analysis was performed at the CDC in Atlanta, GA, USA. These samples were shipped in their bottles via 2-day air freight in Air Sea Containers (Miami, FL, USA) with cold-packs to the Environmental Microbiology Laboratory at the CDC on a weekly basis. Upon arrival at CDC, samples were placed at 4°C until processing. All samples were processed for moisture analysis and *Ascaris* viability within 36 days of collection.

### Temperature Analysis

Prior to sample collection, compost temperature was measured *in situ* at each sample collection site, at the 45-cm depth in the center and corner of the bin using a 60-cm thermometer probe. The probe was rinsed with both 10% bleach and 70% ethanol solutions between readings. Temperature measurements of untreated latrine waste and final compost products were not performed.

### Moisture Analysis

Moisture analysis and calculations were carried out according to Standard Method 2540B [20] using a starting amount of 15 g (wet weight) of each sample. The final two dry weight measurements recorded (exhibiting a difference of <4%) were averaged and reported as a single final dry weight for the sample. Percent moisture was calculated from the final and original weights to convert *E*. *coli* concentrations from a wet weight basis to a dry weight basis.

For the analysis of repeat samples from Bin 1, samples were averaged across locations (center, corner) and across all sampling events to generate a single value for percent moisture over the 14 day period. For the cross-sectional analysis, samples were averaged across locations within each bin. Final compost samples were averaged as well.

### *E*. *coli* Assessment

The protocol for *E*. *coli* assessment was adapted from Eccles et al. [21] and Muirhead et al. [22]. Prior to analysis, each individual sample was thoroughly mixed at LNSP by emptying the entire contents of the 125-mL Nalgene bottle into a large metal bowl and stirring with a large metal spoon for one minute. After mixing, approximately 1 g of compost material (maximizing fecal material over husk material) was added to 10 mL of 0.01 mol L^-1^ phosphate-buffered saline (PBS) and vortexed for one minute to break up clumps. The sample was then diluted 1:10 in 9 mL PBS and vortexed again for one minute. One mL of the suspended sample was added to 99 mL sterile, deionized water in an IDEXX (Westbrook, ME, USA) bottle containing sodium thiosulfate, briefly vortexed, and processed using the standard IDEXX Colilert protocol. Samples were observed under UV light after 24 ± 1 hours of incubation at 37 ± 2.5°C and most probable number (MPN) concentrations of *E*. *coli* were recorded. The amount of *E*. *coli* in center and corner samples was compared for each bin by paired Student’s T-tests in SAS (SAS Corporation, Cary, NC).

### *Ascaris* Viability Assessment

*Ascaris* spp. viability was assessed using a modified version of the US Environmental Protection Agency (USEPA) method for sludge [23]. After being weighed, samples were placed in 4-L beakers with 500 mL of deionized water and incubated overnight at 4°C. Following incubation, samples were blended for one minute, 1L of 1% 7X detergent was added to the homogenized mixture, and the sample was incubated overnight at 4°C. Following the second incubation, samples were blended, sieved using a 20-mesh sieve followed by a 400-mesh sieve, concentrated by flotation with MgSO_4_, and incubated in 4 mL of 0.05 mol L^-1^ H_2_SO_4_ at 25 ± 1°C for 21–28 days.

Incubated samples (100–200 μL; 5–10% sample volume) were examined under a light microscope (Olympus BX51, Center Valley, PA, USA) for viable and/or embryonated *Ascaris* eggs. Because *Ascaris* egg concentrations were low, the incubation solution was not re-suspended prior to microscopic examination in order to maximize the number of *Ascaris* eggs that could be examined per slide and thus percentages of *Ascaris* eggs that were viable and fully embryonated were calculated for each sample. Due to concerns about incubation conditions (presented later) and to allow more conservative estimation of viability, the USEPA viability classification guidelines [23] were modified for this study. *Ascaris* eggs were classified as *unembryonated* if there were no visible signs of cleavage, as *viable* if cleavage had begun (i.e, ≥ 2 cells with intact cell membranes were observed per egg), and as *fully embryonated* if a motile worm was observed. For each cross-sectional sample, 100–200 total *Ascaris* eggs were counted and classified according to these categories. For samples from Bin 1, with repeat observations per location, two slides were observed per sampling event and results were pooled by summing across samples within each classification (total, viable, or fully embryonated) prior to analysis. A total of five samples had insufficient *Ascaris* eggs for analysis (<5 per slide after evaluation of at least two slides) and were designated as “TFTC” (too few to count).

A negative control and a positive percent recovery assay were conducted as quality control measures. Three hundred grams of autoclaved potting soil (Scott’s Earthgro potting soil; Mfg. model #: 72440180, Marysville, OH, USA) was processed according to the USEPA protocol as a negative control. To determine percent recovery, 9 x 10^4^ *Ascaris suum* eggs (Excelsior Sentinel, Inc., Newfield, NY, USA) were added to 156 g of autoclaved potting soil in a 500 mL Nalgene bottle, which was shaken and incubated overnight at 4°C prior to beginning the protocol. The sample was assayed as previously described and *Ascaris* eggs in 85 μL of final sample were enumerated and used to calculate the percent recovery for the method.

## Results

### Temperature Analysis

**Fig 2:**
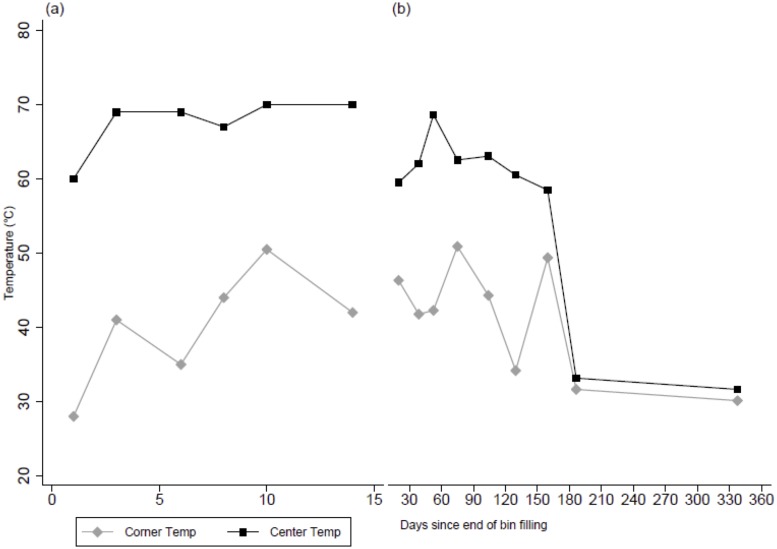
Compost temperatures by time since end of bin filling. Temperature measurements are presented for two locations—the center (dashed line) and the corner (solid line)—of each compost bin. a) Longitudinal temperature measurements over the first 14 days of filling of Bin 1; b) Cross-sectional temperature measurements of bins filled 15–330 days prior to sampling (Bins 2–10).

Over the first two weeks of composting, center temperatures in Bin 1 ranged from 60–70°C. Center temperatures remained at or above 58°C in bins until compost was moved to an open area pile after 6 months (180 days). During the first 6 months of composting, temperatures in the center of bins were consistently higher than temperatures in the corners of the bin, which never reached above 51°C (Fig 2).

### Moisture Analysis

Untreated latrine waste samples had moisture content ranging from 75–82%, with an average of 79%. Compost from Bin 1 averaged 70% moisture over the first two weeks. Compost from Bins 2–10 averaged 67% moisture. In final samples, the moisture content dropped to an average of 45% (S1 Table).

### *E*. *coli* Assessment

**Fig 3:**
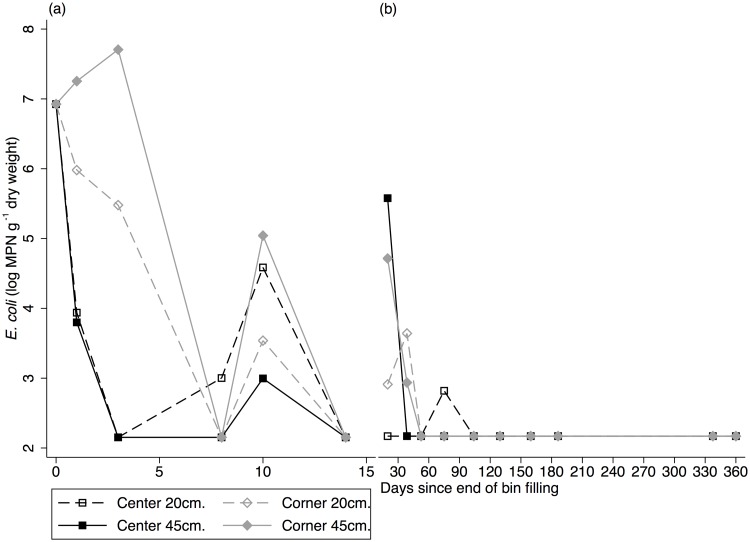
Concentration of *E*. *coli* in compost. Concentration of *E*. *coli* in compost was measured as the geometric mean of multiple dilutions (MPN g^-1^ dry weight, log-transformed). Concentrations were measured in compost samples taken at depths of 20 cm (dashed lines) and 45 cm (solid lines) in center (black, squares) and corner (gray, diamonds) locations. (a) Baseline and Longitudinal Samples: Baseline *E*. *coli* concentration at Day 0 is an average of five untreated latrine waste samples. Additionally, Bin 1 was sampled 5 times longitudinally over the first 14 days of filling. (b) Cross-sectional samples were taken from Bins 2–10, which represented different ages of compost. ‘Final’ compost (represented as 360 days) was sampled twice and averaged.

*E*. *coli* concentrations per gram dry weight of compost are presented in Fig 3. The baseline concentration of *E*. *coli* in untreated latrine waste samples ranged between 10^6^–10^7^ MPN g^-1^ dry weight, which is represented by the average latrine waste sample concentration in Fig 3a at 0 days. *E*. *coli* levels in repeat samples from Bin 1 varied considerably, from below the limit of detection (<142 *E*. *coli* MPN g^-1^ dry weight) to close to 10^8^ MPN g^-1^ dry weight (Fig 3a). Cross-sectional samples from Bins 2–10 show a decrease in *E*. *coli* concentrations over time from 15–120 days of compost age. *E*. *coli* was initially detectable in the intermediate-age cross-sectional bins, but after 75 days, samples from all locations and depths produced results below the limit of detection (Fig 3b), as also noted in Preneta et al. [19]. *T*-tests comparing *E*. *coli* levels at a given depth by location revealed no significant differences between center and corner *E*. *coli* concentrations within a given bin. Overall, a 4–5 log reduction was observed over the sampling period.

### *Ascaris* Viability Assessment

The percent recovery of *Ascaris* eggs using the USEPA method was 71.6%. To determine progress in egg development and cleavage in processed compost samples, a randomly-selected untreated latrine waste (0 days of composting) sample was observed weekly over the course of the 28 day incubation period. Because little development of *Ascaris* eggs was observed in this sample after 28 days of incubation using the USEPA method (incubation in H_2_SO_4_), half the volume of each sample (2 mL) was re-suspended in 0.5% formalin [the incubation solution used in the Bowman et al. [24] protocol]. After 28 subsequent days of incubation in formalin, approximately 25% of *Ascaris* eggs in the representative untreated latrine waste sample were fully embryonated and motion-sensitive to light, and approximately 40% had begun to undergo cleavage. Less than 1% of *Ascaris* eggs incubated in H_2_SO_4_ over the same additional time period (a total of 56 days) had begun cleavage and none were fully embryonated. Thus, the remaining samples were evaluated for viable *Ascaris* eggs 28 ± 2 days after resuspension in formalin.

The results from the microscopic evaluation of *Ascaris* survival are presented in Table 2. We evaluated six untreated latrine waste samples, five of which (83%) contained *Ascaris* eggs in sufficient quantities to be counted. Of these five, an average of 180 *Ascaris* eggs per sample were assessed for viability. Three of the five latrine waste samples (60%) exhibited viable and/or fully embryonated *Ascaris* eggs. Untreated latrine waste samples exhibited the highest average percent of viable (14%, range 0–46%) and fully embryonated *Ascaris* eggs (12%, range 0–42%) among all sample types. Samples from Bin 1 were pooled by sampling location (center: 5 samples; corner: 6 samples) over the 14-day collection period. Three of the five center samples (60%) contained sufficient *Ascaris* eggs for enumeration, while all five of the corner samples contained sufficient *Ascaris* eggs. A total of 361 *Ascaris* eggs were counted in the center pooled sample and 575 in the corner pooled sample. Overall, 0.4% and 1.7% of observed *Ascaris* eggs from Bin 1 corner samples were fully embryonated and viable, respectively, while neither viable nor fully embryonated *Ascaris* eggs were detected in center samples.

**Table 2:** *Ascaris* spp. viability profile.

Bin	Sample Type	Age of Compost	Location	Samples Analyzed	Total *Ascaris* Eggs Analyzed	Viable (%)	Fully Embryonated (%)
0	Untreated	0 days	–	5^†^	900	94 (10.4%)	75 (8.3%)
1	Int.	1, 3, 8, 10, 14 days	Center	3^‡^	161	0 (0.0%)	0 (0.0%)
	Int.		Corner	6	575	10 (1.7%)	2 (0.4%)
2	Int.	2 weeks	Center	1	100	2 (2.0%)	0 (0.0%)
	Int.		Corner	1	102	1 (1.0%)	0 (0.0%)
3	Int.	4 weeks	Center	1	112	0 (0.0%)	0 (0.0%)
	Int.		Corner	1	200	0 (0.0%)	0 (0.0%)
4	Int.	6 weeks	Center	1	122	0 (0.0%)	0 (0.0%)
	Int.		Corner	1	200	1 (0.5%)	0 (0.0%)
5	Int.	8 weeks	Center	1	123	0 (0.0%)	0 (0.0%)
	Int.		Corner	1	TFTC	–	–
6	Int.	12 weeks	Center	1	107	0 (0.0%)	0 (0.0%)
	Int.		Corner	1	200	0 (0.0%)	0 (0.0%)
7	Int.	16 weeks	Center	1	113	0 (0.0%)	0 (0.0%)
	Int.		Corner	1	TFTC	–	–
8	Int.	20 weeks	Center	1	100	0 (0.0%)	0 (0.0%)
	Int.		Corner	1	100	0 (0.0%)	0 (0.0%)
9	Int.	24 weeks	Center	1	117	0 (0.0%)	0 (0.0%)
	Int.		Corner	1	200	0 (0.0%)	0 (0.0%)
10	Int.	44 weeks	Center	1	100	0 (0.0%)	0 (0.0%)
			Corner	1	105	0 (0.0%)	0 (0.0%)
N/A	Final	48–96 weeks	–	2	218	0 (0.0%)	0 (0.0%)

Note: Intermediate samples are abbreviated “Int.”

^†^One sample excluded due to TFTC;

^‡^Two samples excluded due to TFTC

For samples collected from Bins 2–10, an average of 131 *Ascaris* eggs were counted per sample, with only 2 samples (corner samples representing 8 and 16 weeks of composting) having an insufficient numbers to count. No samples from these bins had fully embryonated *Ascaris* eggs, and a maximum of 1% and 2% of *Ascaris* eggs from the corners and centers of the bins, respectively, were viable. No viable *Ascaris* eggs were found in any samples of compost that were 8 weeks or older, as also noted in Preneta et al. [19]. Samples of ‘final’ compost had 109 *Ascaris* eggs counted on average and no viable *Ascaris* eggs were observed in any of these samples. Overall, a greater than 1-log reduction in *Ascaris* spp. egg viability was observed over the sampling period.

## Discussion

We examined die-off of *E*. *coli* and *Ascaris* across time in an EcoSan composting system, under field conditions, in order to evaluate its efficacy in producing products for eventual human handling and agricultural use. We observed variability in both *E*. *coli* concentration and *Ascaris* viability in repeat samples taken over the first two weeks of the composting process. Cross-sectional assessment of compost ranging from 2–96 weeks of age revealed greater than 4-log and 1-log reductions, respectively, for *E*. *coli* and *Ascaris* spp. egg viability within 16 weeks, and remained undetectable after this time. These results are comparable to those of other studies using similar isolation methods to evaluate *E*. *coli* and *Ascaris* inactivation [16, 18, 25].

### Factors Affecting Microorganism Die-Off

Microorganism die-off in this off-site compost system occurred more rapidly than previously observed in in-latrine EcoSan field studies [3, 8, 12–14, 26], but on a similar timescale to those observed in other off-site systems [25, 27]. *E*. *coli* and fecal coliforms can survive for months in soil or sludge at temperatures of 20–30°C [4], but can be inactivated within minutes to days by exposure to temperatures upwards of 50°C [28]. *Ascaris* eggs have been shown to survive in sludge and compost for 1–4 months at ambient temperatures of 20–30°C, but survival can decrease to a few hours to two weeks at elevated temperatures of 40–50°C [3, 17, 18, 27, 29].

Despite substantial differences in temperature profiles between the corner and center of the bins (Fig 1), we observed similar *E*. *coli* inactivation profiles in both locations (Fig 2). However, viable *Ascaris* eggs appeared to survive longer in corner samples (up to 6 weeks) than in center samples (up to 2 weeks). While we measured both temperature and microbial concentration and viability in the compost bins, we could not differentiate between the effects of temperature and time on microorganism die-off.

In the current evaluation, latrine waste (fecal waste + sugar cane husk) was consolidated from multiple latrines at a central location. Moving the waste out of the latrines and into large volume bins likely increased the temperatures of the compost to levels not observed in previous analyses of double-vault composting latrines. Thermophilic composting in on-site (in-latrine) EcoSan latrine models has been largely unsuccessful in reaching microorganism die-off at levels close to or below the standard limits of detection for indicator testing. In the only EcoSan study showing in-latrine *Ascaris* spp. inactivation on a shorter timescale (40 days) than that observed here, dessication, not thermophilic composting, was the method of inactivation, with ash and/or lime added to the compost to increase pH in the latrine [16].

Other off-site systems have shown similar time profiles for microbe inactivation. Pourcher et al. [25] documented longitudinal maximum temperature profiles and moisture content in compost bins of similar size and using similar organic material (straw) to those evaluated here. Temperatures were comparable to the center temperatures that we observed across the first 4 months and 2–5 log reductions were observed in *E*. *coli* throughout the compost pile during this time period. However, samples of *E*. *coli* did not reach undetectable levels until the final reading at 7 months and *Ascaris* die-off was not evaluated. Jensen et al. [27] experimentally evaluated *Ascaris suum* die-off in double-vault composting latrine waste allowed to compost outside of the latrine, and found 97% within 88 days and >99% die-off of *Ascaris* eggs within 117 days of storage. *E*. *coli* was not evaluated. In both of these studies, compost was mixed to increase aeration in the compost pile, while the system evaluated here did not incorporate compost mixing, yet achieved similar results.

The addition of urine during the first 2–3 months of processing in the composting system evaluated in this study could have increased the rate of microorganism die-off, including that of *Ascaris*, through elevation of ammonia levels in compost. Ammonia content has been noted as the most critical parameter in the die-off process for *Ascaris* ova in combined (feces and urine) latrine sludge [27]. There is a growing body of literature regarding the efficacy of urine in inactivating microbes in feces from UDTs [30–33]. Addition of urine to feces has been shown to yield *Ascaris* inactivation within 30 days under ambient temperatures (24–34°C) and in the absence of drying materials [30], similar to die-off times observed here. Die-off times in the literature have been observed with as little as 4.7–18.0 g L^-1^ total ammonia nitrogen content, as calculated from mixing ratios of 0.5–1L urine to 0.2 kg feces [30, 33]. Accordingly, the addition of approximately 190 L of urine to each compost bin in the current study may have facilitated *Ascaris* spp. inactivation. However, no urine was added to Bin 1 during the sampling period, suggesting that heat was the primary mechanism for the inactivation observed during the first two weeks of composting.

### Study Limitations

This study was not conducted to evaluate the agricultural quality or adequacy of the compost produced by the EcoSan system. The focus of the study was to determine microbial die-off to evaluate the efficacy of the composting process under field conditions. The limited sample number and analytical parameters used in this study do not provide comprehensive data for characterizing the overall or long-term safety of final product from the EcoSan system.

Given that our study was a field evaluation under naturally-occurring conditions, we did not regulate the *Ascaris* content of the compost. Initial *Ascaris* viability in the compost was low compared to other indigenous *Ascaris* studies [18, 34], we therefore used percent viability of *Ascaris*, rather than *Ascaris* concentration, as our outcome and attempted to standardize the range of *Ascaris* eggs observed per sample. This low viability could have been due to heat inactivation in the collection barrels, where new latrine waste was held for 1–5 days before being evaluated here as untreated latrine waste samples. While we did not measure temperature within the untreated latrine waste barrels, we did observe that they were warmer than ambient temperature at the time of emptying into the bins and collecting samples. Increases of even 5–10°C relative to ambient summer temperatures during this lag period could have resulted in 40–50°C temperatures inside the barrels, more than adequate to begin inactivation [18, 27, 29, 35]. Future in-barrel temperature measurements would be useful to evaluate further describe this potential inactivation. While we incubated samples for *Ascaris* viability testing at room temperatures (25 ± 1°C) that were within the range recommended by established *Ascaris* viability test methods [23, 24], we recognize that *Ascaris* culture may be improved by incubating at higher temperatures (e.g., 28–30°C) reported by other researchers [36, 37]. Additionally, while this study used fresh latrine waste samples containing *Ascaris* eggs as its positive control, we would recommend that a stock solution of *Ascaris* eggs be used as a positive control in future research to monitor the adequacy of incubation conditions for embryonation of viable *Ascaris* eggs.

Due to logistical limitations, we did not measure pH and ammonia content and we are therefore unable to specifically evaluate the effect of urine addition on microbial die-off in the compost. Future EcoSan studies should include measurement of these parameters if the composting process includes urine addition. Given the short sampling period in this study, we were only able to collect single samples per location and depth for bins older than two weeks. Thus, the data were insufficient to construct a longitudinal model evaluating factors such as temperature, time, and moisture content individually. While we were unable to follow the same biosolid material longitudinally in the year prior to the study, there was no reported change in the health status of the population using the latrines, no latrines were built in new geographic regions, and no large groups entered or left the population served by the latrines during this time period. Thus, we assumed the microbiological profile of the latrine waste entering the bins at the time of study was consistent with the general profile of latrine waste entering the bins over the previous months.

Our study was unique in its ability to evaluate microorganism inactivation in composted biosolids of up to almost two years in age using a combination of sampling of untreated latrine waste, repeated sampling of solids in the early stages of the composting process, and cross-sectional samples of compost of varying ages. Both the composting process and evaluation sampling methods used are applicable to other resource-poor settings. The composting process consisted of locally-available materials and did not require machinery or technology outside of trucks to transport the compost and barrels and bins to store the compost. Sugarcane husk is widely available throughout Haiti and in other developing communities. Taken together, these methods could provide a basis for large-scale, low-technology composting operations with consistent monitoring and evaluation.

### Evaluation and Compliance Standards

The Haitian government has recently established standards for microorganism content for biosolid material reuse [38]: < 10^2^ *E*. *coli* (CFU vs. MPN not stipulated) per gram wet weight and 0 viable helminth eggs per 1.5 g wet weight. While these standards appear stricter than those of the USEPA (see below), it is difficult to directly compare our results to them, given the variability in moisture content of the sludge examined. The USEPA microbiological guidelines for the reuse and disposal of biosolid material are divided into “Class A” (unrestricted use by the public) and “Class B” (restrictions on application on lands accessed by the public and/or used for growing edible crops) standards. To be considered “Class A,” biosolids must have <1000 MPN fecal coliforms per gram total dry solids (TS) and <1 viable helminth eggs per 4 grams TS. “Class B” biosolids allow <2 × 10^6^ MPN g^-1^ TS [39]. The compost evaluated here reached <1000 MPN *E*. *coli* g^-1^ TS and appeared to approach <1 viable helminth egg per 4 grams TS within 8 weeks. However, our results cannot directly compare compost quality to USEPA standards, as *E*. *coli* is generally recognized as underestimating fecal coliform concentrations because it is only one of many members of the fecal coliform group [40]. In addition, while we processed compost samples for *Ascaris* species using the USEPA isolation method, because of time and personnel constraints, only a portion (100–200 μL or ~10%) of each final sample was evaluated for the presence of *Ascaris* eggs. Therefore, for the majority of samples we were unable to directly compare our results to EPA “Class A” guidelines of <1 viable helminth eggs per 4 g TS.

To ensure that organizations implementing EcoSan projects can easily monitor their systems and ensure the safety of their final compost products, it is necessary to have microbiological evaluation methods available that can be simply, reliably, and effectively performed in field or resource poor settings. The modified Colilert method used here to detect *E*. *coli* is a relatively accessible system that can be performed by technicians with little specialty training; however, it remains resource- and cost-intensive. Furthermore, while this study demonstrated similar inactivation periods for *E*. *coli* and *Ascaris* spp., this finding is not well supported in the literature, as *E*. *coli* is a less thermotolerant organism than *Ascaris* spp. Thus we would recommend continued incorporation of helminth testing in future evaluations. However, the USEPA method for evaluating *Ascaris* spp. viability in biosolids is limited by its technical difficulty, reagent requirements, and extended incubation period. Alternative analytical methods that do not require such long incubation times or labor-intensive microscopy are needed.

Results of previous field studies on aerobic Ecosan composting latrine waste have been inconclusive regarding which composting parameters—e.g., time, temperature, aeration, pH, moisture content—are most important to monitor as proxies for the efficiency of microorganism inactivation. Further studies of the comparative survival of *E*. *coli*, *Ascaris*, and other fecal microbes relative to these compost parameters would be useful to supplement our study results. Furthermore, they would provide context to support the use of *E*. *coli*, temperature, moisture, and/or potential proxy organisms for *Ascaris* as more feasible and cost-effective process and microbial indicators for monitoring compost systems, especially in resource-poor regions of the world.

## Supporting Information

S1 Table Moisture content data for compost samples. (DOCX)

S1 Data Spreadsheet containing *E*. *coli*, *Ascaris*, temperature and moisture content data for EcoSan study. (XLSX)
