# Supplementary material for: Ascaris and Escherichia coli Inactivation in an Ecological Sanitation System in Port-au-Prince, Haiti
Source: PLoS One. 2015 May 1;10(5):e0125336. doi: 10.1371/journal.pone.0125336 (PMC4416818; doi:10.1371/journal.pone.0125336)
Supplement: S1 Table — (DOCX) [file pone.0125336.s001.docx]

**Supporting information**:

| Table S1: Moisture Content | | | |
| --- | --- | --- | --- |
| Bin | Sample Type | Age of Compost | Moisture content (%) |
| 0 | Untreated | 0 days | 79.1 |
| 1 | Intermediate | 1, 3, 8, 10, 14 days | 70.4 |
| 2 |  | 2 weeks | 57.1 |
| 3 |  | 4 weeks | 71.5 |
| 4 |  | 6 weeks | 72.0 |
| 5 |  | 8 weeks | 61.9 |
| 6 |  | 12 weeks | 66.8 |
| 7 |  | 16 weeks | 71.1 |
| 8 |  | 20 weeks | 70.3 |
| 9 |  | 24 weeks | 66.9 |
| 10 |  | 44 weeks | 66.3 |
| N/A | Final | 48 – 96 weeks | 44.7 |
